# GP access for inclusion health groups: perspectives and recommendations

**DOI:** 10.3399/BJGPO.2024.0021

**Published:** 2024-07-10

**Authors:** Aaminah Verity, Victoria Tzortziou Brown

**Affiliations:** 1 Wolfson Institute of Population Health, Queen Mary University London, London, UK

**Keywords:** general practice, health policy, health inequities, access

## Abstract

**Background:**

General practice has seen the widespread adoption of remote consulting and triage systems. There is a lack of evidence exploring how inclusion health populations have been impacted by this transformation.

**Aim:**

This study aimed to explore the post-pandemic GP access for inclusion health populations, through the lens of those with lived experience, and identify practical recommendations for improving access for this population.

**Design & setting:**

A mixed-methods study exploring the direct experience of people from inclusion health groups trying to access GP care in 13 practices in east London.

**Method:**

A mystery shopper exercise involving 39 in-person practice visits and 13 phone calls were undertaken. The findings were reflected on by a multidisciplinary stakeholder group, which identified recommendations for improvements.

**Results:**

Only 31% of the mystery shopper visits (*n* = 8) resulted in registration and the offer of an appointment to see a GP for an urgent problem. None of the mystery shoppers was able to book an appointment over the phone but *n* = 10/13 felt that they would be able to register and make an appointment if they followed the receptionist's instructions. Most mystery shoppers felt respected, listened to, and understood the information provided to them. Just under half of the practices (46%, *n* = 6) received positive comments on how accessible and supportive their spaces felt. Practice- and system-level recommendations were identified by the stakeholder group.

**Conclusion:**

Ongoing GP access issues persist for inclusion health populations. We identified practice- and system-level recommendations for improving access for this vulnerable population.

## How this fits in

To date, there has been little published research exploring the impact of remote consultation and triaging on GP access for inclusion health populations. Our study highlights the considerable variability in GP practices’ ability to register and book appointments for patients from inclusion health backgrounds.There is a need for practical resources assisting practices to assess and improve their access for these populations. There is also a need for an embedded culture of proactive identification of vulnerable patients and tailored care provision.

## Introduction

General practice in the UK has undergone one of the biggest transformations since its inception with the widespread adoption of online and remote consulting and triage system.^1,2^ While facilitating a successful response to the initial risk of COVID-19 transmission, these new systems of working need to be refined and evolve according to patient needs and within the context of financial and staffing constraints.

There have been several studies exploring the impacts of how primary care has adapted,^2–8^ although with limited exploration of how these changes have affected health inequalities. A relevant systematic review^9^ found that studies were particularly lacking for people from inclusion health populations who are traditionally socially excluded, experience stigma and discrimination, and already struggle to access and engage with health care. Studies on the barriers such populations experience when trying to access primary care since the pandemic have not been translated into tangible recommendations for practices to address these issues.^10–13^

This study aimed to explore the experiences and perspectives of people from inclusion health groups and identify practical recommendations for improving access using co-production methodology.

## Method

The first part of this study has already been published.^14^ It used semi-structured interviews to explore the perspectives of people from inclusion health groups on remote consulting and triage-first models of general practice.

This report presents the two other parts of the study, which included a mystery shopper exercise aiming to understand the true experience of inclusion health populations attempting to access care under the current system and a series of workshops with a wider stakeholder group where the findings were presented, key themes were identified, and recommendations were developed.

### Study sites

All primary care networks (PCNs) in Newham and Tower Hamlets were invited to participate and three PCNs (13 practices in total) were recruited into the study.

### Mystery shopper exercise

A mystery shopper design enables the collection of service performance information^15^ and can be helpful for studying healthcare provider behaviour in a first-hand way while minimising observation bias.^16^

The mystery shopper exercise was led by Groundswell,^17^ a charity that brings together insights from people with experience of homelessness. The methodology and materials were co-produced with peer researchers and volunteers with lived experience of homelessness and of living in other marginalised situations (Experts by Experience [EbE] group).

A review of existing resources, guidelines, and standards on GP access was undertaken. This summarised the access guidance from publications by: statutory organisations, comprising NHS England (NHSE), the Care Quality Commission (CQC), National Institute for Health and Care Excellence (NICE), and British Medical Association (BMA); and voluntary and third-sector organisations, comprising Doctors of the World (DOTW), Groundswell, and Pathway. This guidance was summarised in a table with input from inclusion health specialists (Supplementary Information S1). This review informed the development of a framework that included a range of indicators that assessed the accessibility of a general practice for inclusion health populations (Supplementary appendices 2–4).

Groundswell recruited and trained a group of volunteers to become mystery shoppers. These volunteers had prior experience of homelessness.

### Practice visits

The EbE group were supported to develop mystery shopper personas for the practice visits through workshops with Cardboard Citizens, a charity and theatre company. Personas differed in terms of demographics and type of social exclusion (Table 1).

**Table 1.** Mystery shopper personas and accessibility issues

Character	Age in years, sex	Ethnic or national identity	Housing status	ID and proof of address	Additional challenges
Steve	51, male	White British	Rough sleeping	No proof of address, supermarket clubcard only	Low charge on phone, difficulties with reading and writing
Marcus	55, male	White British	Sofa surfing	No proof of address, supermarket clubcard only	Has internet access for online consultation
Seb	40, male	Polish	Rough sleeping	None	Struggles with English, low charge on phone
Christina	35, female	Black African British	Staying with friend	None	Mobility issues
Annabel	30, female	White European	Staying with friend	None	Fleeing domestic violence, no internet access, and doesn’t take calls from private numbers

ID = identification.

Mystery shoppers attended the GP surgeries requesting an appointment for a problem requiring urgent medical attention (either cough with blood in sputum and night sweats, or change in bowel habit plus weight loss). Mystery shoppers were not in distress or acute mental or physical health crisis.

PCN clinical directors were asked to notify practice managers about the mystery shopper exercise and give a timeframe of 2 weeks within which the visits would take place. In total, 26 visits (two per practice) were undertaken over a 2-week period.

### Telephone interactions

A separate telephone registration and appointment attempt was made by mystery shoppers at another time. Mystery shoppers stated they were sofa surfing at a friend’s house nearby, using a friend’s phone with no internet access. They stated that they wanted to register and get a new prescription for anti-epileptic and diabetic medication because their medication was running out in 3 weeks. Mystery shoppers were instructed to call practices on the main practice number three times outside of the peak hour of 8.00 am–9.00 am and record the practices’ responses.

### Practice accessibility assessments

Mystery shoppers performed a separate assessment of the practices’ accessibility by visiting each practice and its website, without interacting with staff.

After each of the above activities, Groundswell researchers met with the mystery shoppers and completed the relevant surveys (Supplementary appendices S2–4) detailing their experiences. The feedback was then analysed using descriptive methodology.

### Stakeholder group workshops

The results of the semi-structured interviews^14^ and mystery shopper exercise were reflected on by a stakeholder group to draw key themes and recommendations.

The stakeholder group included EbEs, mystery shoppers, PCN directors, GPs, and practice receptionists and managers from the practices that took part in the project, healthcare commissioners, and Groundswell and Pathway representatives.

Before the meetings, the research team reviewed the initial study findings and identified key issues to present at the workshop.

Three 2-hour online workshops were hosted by the study authors and were attended by 20–25 participants. Workshops were held on Zoom and were 3 weeks apart to give participants time to reflect on the previous conversations. Members of the research team acted as facilitators. Google Jamboard was used to live-capture minutes from the workshops and promote discussion in breakout groups. Responses were audio-recorded and transcribed to help with the analysis of the data and draw out the key recommendations. A summary of each workshop's findings was drafted and sent to all attendees who were able to provide comments after which the report was finalised.

## Results

### Mystery shopper exercise: practice visits

**Figure 1.**
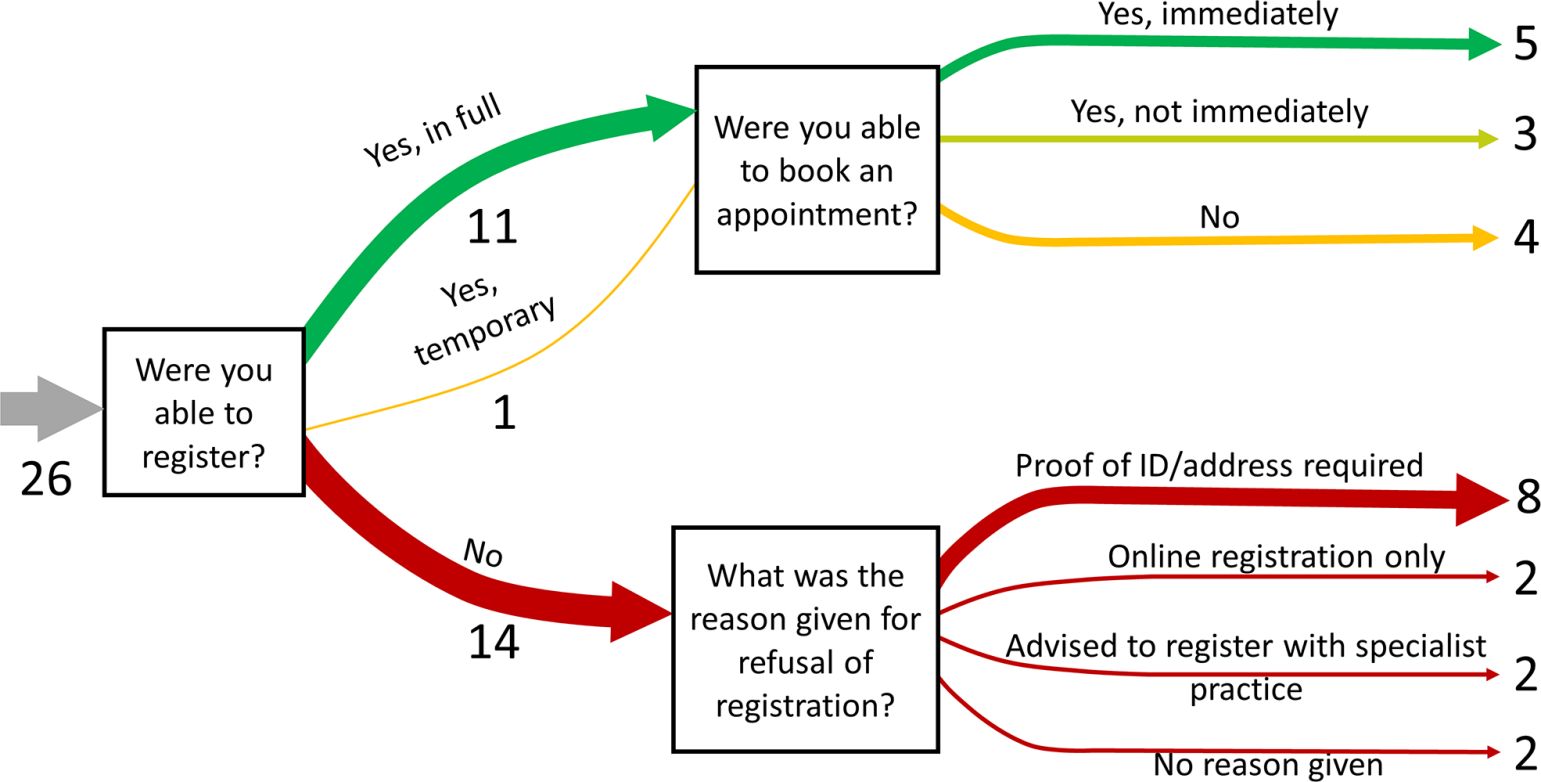
Outcomes in registration and appointment booking from mystery shopper visits

All mystery shoppers were able to walk in and speak to practice staff. The results of the registration attempts by the mystery shoppers during the 26 practice visits are presented in Figure 1. The results show that 31% of the visits (*n* = 8) resulted in registration and the offer of an appointment to see a GP. Almost half of the visits 54% (*n* = 14) ended with registration refusal and most of these refusals (57%, *n* = 8) were owing to mystery shoppers’ inability to provide proof of identification (ID) or address.

Only in four of the visits were mystery shoppers offered the option of using the practice as their proxy address. In *n* = 7/26 visits, mystery shoppers were given choices about their communication preferences (for example, email, text, and so on). In *n* = 9/26 visits mystery shoppers were signposted to specialist homeless services and in *n* = 4/26 visits they were signposted to other services. In *n* = 3/26 visits mystery shoppers were asked for the reason of the appointment request. Only one out of the eight mystery shoppers who managed to get an appointment was asked their preferences on the time and type of the appointment.

The results of the experiences of mystery shoppers during the visits are presented in Table 2.

**Table 2.** Mystery shoppers’ perceptions of how they were treated by reception staff

	Strongly agree % (number of visits)	Agree % (number of visits)	Neither agree nor disagree % (number of visits)	Disagree % (number of visits)	Strongly disagree % (number of visits)
Practice visits (*n* = 26)					
I was treated with respect by staff	34.6% (9)	46.2% (12)	7.7% (2)	7.7% (2)	3.8% (1)
I felt listened to by staff	26.9% (7)	46.2% (12)	11.5% (3)	11.5% (3)	3.8% (1)
I was understood by staff	11.5% (3)	42.3% (11)	11.5% (3)	26.9% (7)	7.7% (2)
I understood the information I was given	42.3% (11)	50.0% (13)	7.6% (2)	0.0%	0.0%
Staff were motivated to help me	15.4% (4)	42.3% (11)	3.8% (1)	23.1% (6)	15.4% (4)
Telephone interactions (*n* = 13)					
I was treated with respect by staff	23% (3)	46% (6)	23% (3)	8% (1)	0%
I felt listened to by staff	15% (2)	38% (5)	31% (4)	8% (1)	8% (1)
I was understood by staff	15% (2)	23% (3)	31% (4)	31% (4)	0%
I understood the information I was given	15% (2)	69% (9)	8% (1)	8% (1)	0%
Staff were motivated to help me	23% (3)	23% (3)	31% (4)	8% (1)	15% (2)

Most mystery shoppers felt respected and listened to, and understood the information provided to them. However, nearly 40% felt that staff were not motivated to help (Table 2).

### Mystery shopper exercise: telephone interactions

The mystery shoppers made 13 attempts, one per practice, to ask for support to register over the phone. All the phone lines were at a standard rate. All callers got through to a receptionist but three of them had to wait for longer than 30 minutes.

In *n* = 11/13 telephone interactions, mystery shoppers were told no ID or proof of address was required for registration. In seven cases they were asked for the details of their previous general practice. None of the mystery shoppers was offered the option of using the practice as their proxy address and none was able to register over the phone. Two mystery shoppers were signposted to other local services. In three cases, mystery shoppers were asked for the reason of the appointment and advised to get a printout of their medication from their previous GP. None of the mystery shoppers was able to book an appointment over the phone but *n* = 10/13 felt that they would be able to register and make an appointment if they followed the receptionist's instructions.

The results on the mystery shopper telephone experience are presented in Table 2. While most mystery shoppers felt respected and listened to, and understood the information provided to them, just under 50% felt staff were motivated to help them.

### Mystery shopper exercise: practice accessibility assessment

One visit per practice was made to assess how accessible the practice felt to someone from an inclusion health background.

The majority of practices (70%, *n* = 9) clearly displayed their opening times and all practices seemed to be accessible to those using a wheelchair. Only 38% (*n* = 5) of practices had information in a different language available or advertising availability of interpreter services.

Thirty-eight per cent (*n* = 5) of practices had information pertaining to support organisations or services for people from inclusion health backgrounds. Just under half of the practices’ space (46%, *n* = 6) attracted positive comments from the mystery shoppers who seemed to value a friendly atmosphere with comfortable seating, walls with information or paintings, signs indicating the practice is a safe surgery, and short queues of people waiting at reception.

### Co-production workshops

On average, 20 people attended each of the three online workshops representing general practices and PCNs, NHS commissioners, EbE, and health inclusion organisations.

#### Workshop 1: Reviewing the study findings and discovering themes

In the first workshop, the study findings were presented to the participants who reflected on the need for recommendations for improvements. Two key types of recommendations were identified: practice-level changes; and system- and advocacy-level changes. It was agreed that it was important to understand the enabling factors for good practice and learn from practices that performed well in the mystery shopper exercise.

#### Workshop 2: Building practice-based recommendations

Representatives from practices which performed well were invited to attend the second workshop that explored practice-level recommendations. Practices with good performance in the mystery shopper exercise said that they talked about the DOTW Safe Surgeries principles at every induction for new members of staff, they called reception staff 'care navigators', indicating their role was to ensure patients get to the right type of care, had easy access to a senior staff member for answering queries, and used an automated registration system to ensure reception capacity for supporting vulnerable patients.

Drawing on the above and on the summary guidance presented in Supplementary appendix S1, the workshop resulted in a list of practice-level recommendations, which are presented in Table 3.

**Table 3.** Practice-level recommendations drawn out from co-production workshop

Domain	Recommendation
Communication with patients	Psychologically informed practice waiting rooms that are welcoming with clear signposting of available support
	Clear and consistent signage on opening hours, how to register and access appointments, and on practice processes including what 'triage' means
	Easy access to interpreters at every phase of the patient interaction, including at reception
Receptionists skills and training	Rebranding receptionists as 'care navigators'
	Education on inclusion health, vulnerability and the human cost of refused registrations and poor access, for example, by using resources such as the EbE film *Less?* ^a^
Digital inclusion	Devices and free Wi-Fi in surgery waiting rooms with support from practice staff (or EbEs or peers) to learn how to use online systems
	Linking with local organisations providing digital inclusion education so they can provide training on NHS online systems to service users
	Review of online consultation tools to ensure these are fit for purpose and easy to navigate
Tailored options to care	Identify and flag individual patient needs, including those patients who may need more support owing to vulnerability
	Walk-in, telephone, and online options for registration and appointment booking
	Offer continuity of care to patients with vulnerabilities and/or complex needs

^a^ *Less?* A film of personal stories and journeys to health from people who have experienced and overcome homelessness. Accessible at: https://journeystohealth.co.uk/.

EbEs = Experts by Experience.

#### Workshop 3: Advocacy and system change recommendations

The third workshop focused on identifying system-level recommendations and advocacy opportunities for improving access to GP care for inclusion health populations. These are presented in Table 4.

**Table 4.** Advocacy and system change recommendations to improve access to general practice for inclusion health populations

Domain	Recommendation
National support for general practices on access and registration	A well-communicated, easily accessible centralised online registration tool that does not require proof of ID or address and that operates in addition to face-to-face and telephone registration options
	Development of a central hub or telephone support line for patients to receive registration support and escalate concerns if they experience challenges with registration
	Communication support tools for practices on registration and appointment booking; step-by-step guides on how to register and access general practice
	Clarification of the role of general practice in emergencies and what are appropriate waiting times for non-urgent issues
General practice funding or contracting	Additional practice funding for registering and caring for patients from inclusion health groups
	National definition and coding of 'vulnerability' and inclusion health groups to ensure consistency and data availability
	Development of relevant incentives or quality markers that can promote access and quality of care for people from inclusion health groups with the aim of tackling health inequalities
Staff roles, recruitment, and retention	Consideration of changing the name and role of practice receptionists to care navigators
	Development and evaluation of recruitment and retention schemes for GPs and other practice staff in areas of high deprivation and health inequalities
Training and education	Inclusion health training of clinical and non-clinical practice staff, including receptionists and practice managers
	Collation and dissemination of training resources on inclusion health to be used as part of practice staff induction training
	National training opportunities on trauma-informed care and practice support or tools to create psychologically informed environments

ID = identification.

## Discussion

### Summary and comparison with existing literature

To date, there has been little published research exploring the impact of remote consultation and triaging on inclusion health populations’ ability to access and effectively navigate GP care.^5,12,13^ This mixed-methods study provides an analysis of the GP access issues faced by inclusion health populations and identifies solutions that can help mitigate these challenges.

The mystery shopper exercise highlights the considerable variability in practices’ ability to register and book appointments for patients from inclusion health backgrounds. Variation in general practice is not new; a multitude of drivers^18–21^ and access issues for inclusion health populations are longstanding, particularly around registration without proof of ID or address.^22–27^ A recent 'deep dive' into understanding the barriers for GP registration^28^ has helped to elucidate some of the reasons behind the ongoing high rates of registration refusals, a phenomenon also observed within our study. Two of our mystery shopper visits were declined owing to the adoption of online-only pathways for registrations, which have become more prevalent since COVID-19. Our study confirms concerns about reduced access owing to the widespread adoption of digital and remote technologies without considering those facing digital exclusion and other barriers.^6,13,29–31^

When discussing how to change cultures and win 'hearts and minds', sharing the human cost of refused registrations and poor access to GP care for inclusion health groups and amplifying the voices of EbEs was considered an important recommendation of this study. Other recommendations for practices included the need for better communication on how different triage systems operate, explaining the different access pathways, and maintaining a walk-in option for those experiencing language or digital exclusion. Also highlighted were the need for increasing practice staff awareness of the relevant NHS England policies around registration for all, ^31–33^ and the embedding of care principles that allow clinical care to continue while registration is being processed. However, it was also acknowledged that, despite a renewed commitment post-COVID-19 to tackle health inequalities,^34^ there are limited practical resources for assisting practices ^35,36^ to assess and improve their access for these populations.

Hence, our study also generated a series of recommendations that need to be implemented at national level. These included the need for national guidance and tools that promote better consistency on the implementation of triage and appointment booking systems. Greenhalgh *et al* ^5,31^ have produced a framework exploring the complexity of deciding when remote consulting is most appropriate, highlighting many system-, patient-, and practitioner-level factors. Such evidence can assist towards producing practical tools for practices to improve their triage and remote consulting policies.

We found that in practices that facilitated urgent appointments and swift registrations for our mystery shoppers, there was an embedded culture of proactive identification and prioritisation of vulnerable patients. Although there is relative consensus on who is vulnerable within health care, with inclusion health populations clearly agreed^37–40^ and some direction from NHSE and CQC,^41–45^ it can be challenging for practices to collate, implement, and embed these recommendations when coming from disparate sources. There is a need for practical guidance summarising the key quality indicators for access for inclusion health groups.

Furthermore, there is a need for the recognition that caring for inclusion health populations requires more time and resource. Evidence has shown that, once weighted for need, practices serving more deprived populations receive around 7% less funding per patient than those serving more affluent populations.^46^ In addition, general practices in deprived areas have on average 14.4% more patients per fully qualified GP.^47^ The principle of proportionate universalism needs to be applied to the resourcing of general practice. Additional funding and evidence-informed staff recruitment and retention initiatives are required in order to ensure that practices have the time and capacity to care for the populations with the greatest needs.

### Strengths and limitations

Assessing the experiences of access to primary care can be very challenging and often relies on patient surveys with variable response rates. Such data do not necessarily include the voice of service users who do not get beyond the first barrier to access. By using mystery shopping as a research tool, this study provided detailed insights and feedback on GP registration and access from a group of service users that is not usually represented in patient satisfaction surveys.

The focus of the study was 13 practices across three PCNs in east London. Given that all practices were in the same geographical area, it is possible that the findings are not generalisable. However, given the significant variation among practices and the fact that similar barriers have been reported in other studies,^11^ we would expect similar findings across other geographical areas.

### Implications for research and practice

Our study produced a series of practice- and system-level recommendations that can assist towards improving GP access for inclusion health populations. There is a need for implementation and evaluation studies that can identify the best way of embedding these recommendations and assess their impact.

In addition, in view of the value of continuity for many of the study participants, there is a need for more evidence on the best ways of facilitating timely access while maintaining continuity for this cohort of patients.

## Data

The dataset relied on in this article is available from the corresponding author on reasonable request.
